# Increases in Genetic Diversity of Weedy Rice Associated with Ambient Temperatures and Limited Gene Flow

**DOI:** 10.3390/biology10020071

**Published:** 2021-01-20

**Authors:** Hua Kong, Zhi Wang, Jing-Yuan Guo, Qi-Yu Xia, Hui Zhao, Yu-Liang Zhang, An-Ping Guo, Bao-Rong Lu

**Affiliations:** 1 Key Laboratory of Biology and Genetic Resources of Tropical Crops, Hainan Key Laboratory for Biosafety Monitoring and Molecular Breeding in Off-Season Reproduction Regions, Institute of Tropical Bioscience and Biotechnology, CATAS, Haikou 571101, China; konghua@itbb.org.cn (H.K.); guojingyuan@itbb.org.cn (J.-Y.G.); xiaqiyu@itbb.org.cn (Q.-Y.X.); zhaohui@itbb.org.cn (H.Z.); zhangyuliang@itbb.org.cn (Y.-L.Z.); 2 Ministry of Education, Key Laboratory for Biodiversity Science and Ecological Engineering, Department of Ecology and Evolutionary Biology, Fudan University, Songhu Road 2005, Shanghai 200438, China; 18110700017@fudan.edu.cn

**Keywords:** gene flow, genetic divergence, increased diversity, temperature effect, temporal isolation, weed

## Abstract

**Simple Summary:**

Increased genetic diversity in plants is probably associated with greater ambient temperatures. To test this hypothesis, we studied genetic diversity and differentiation of weedy rice populations occurring in the early- and late-season rice cultivation fields in Leizhou of southern China. Data collected from 10-year climatic records showed a higher average temperature in the late rice-cultivation seasons than in the early rice-cultivation seasons. Results obtained based on 27 SSR (simple sequence repeat) loci indicated greater genetic diversity in the late-season weedy rice populations, in addition to the considerable genetic differentiation between the early- and late-season weedy rice populations. We therefore conclude that a higher ambient temperature might be an important factor to promote the formation of genetic diversity in the late-season weedy rice populations.

**Abstract:**

Hypotheses regarding the association of increased species or genetic diversity with gradually warmer regions as a global pattern have been proposed, but no direct and solid experimental data are available to approve the association between plant genetic diversity and ambient temperatures. To test the diversity-temperature hypothesis, we studied genetic diversity and genetic differentiation of weedy rice (*Oryza sativa* f. *spontanea*) populations occurring naturally in early- and late-season rice fields that share nearly the same ecological conditions but with slightly different temperatures. Data collected from 10-year historical climatic records indicated a ~2 °C higher average air temperature in the late rice-cultivation seasons than in the early seasons. Results based on molecular fingerprints of 27 SSR (simple sequence repeat) loci showed a higher level of genetic diversity in the late-season weedy rice populations than in the early-season populations. In addition, a positive correlation was detected between the increased proportion of genetic diversity (Δ*H_e_*) and genetic differentiation among the weedy rice populations, suggesting limited gene flow. Therefore, we conclude from this study that increased genetic diversity in the late-season weedy rice populations is probably caused by the higher ambient temperatures. This finding provides evidence for the possible association between genetic diversity and ambient temperatures.

## 1. Introduction

Biodiversity plays an important role in maintaining the balance of the biosphere and sustaining human livelihood [1,2]. To address the questions regarding how biodiversity is created and maintained still remains challenging [3]. It is generally considered that biodiversity is distributed heterogeneously around the globe due to the tremendous differences in the ecological habitats where organisms inhabit. Such differences include temperature variation with latitudinal gradients [4,5,6,7], altitudinal gradients [8], and vertical gradients in the oceans [9]. To determine the reason concerning how such patterns are formed has long been a hot research topic in ecology and evolution fields [5]. A veritable explosion of studies has focused on the large-scale spatial patterns and species levels of biodiversity [10,11,12,13]. However, only a few studies focused on the examination of associations between the temperature gradient and biodiversity at the genetic level within populations or species [14,15].

Genetic diversity is the fundamental sources of biodiversity at the genic level [16,17]. The major task of genetic diversity is to quantify the magnitude of genetic variability within a given population or species. Theoretically, the creation of genetic diversity results from the ability of a population or species of organisms to respond to the changes of the environmental factors [18,19], such as climate and temperature. For example, the relationships between temperature and mutation rates have been well-described in many genetic and molecular studies [20,21]. There is a hypothesis that higher temperatures can promote higher metabolic and mutational rates, which, as a consequence, may increase biodiversity, including genetic diversity [4,22,23]. In other words, this hypothesis emphasizes that genetic diversity could become higher in warmer regions than in the cold regions, which is referred to as “diversity-temperature hypothesis” in this article. However, no solid experimental data are available to approve such a correlation between genetic diversity and the ambient or habitat temperature.

**Figure 1:**
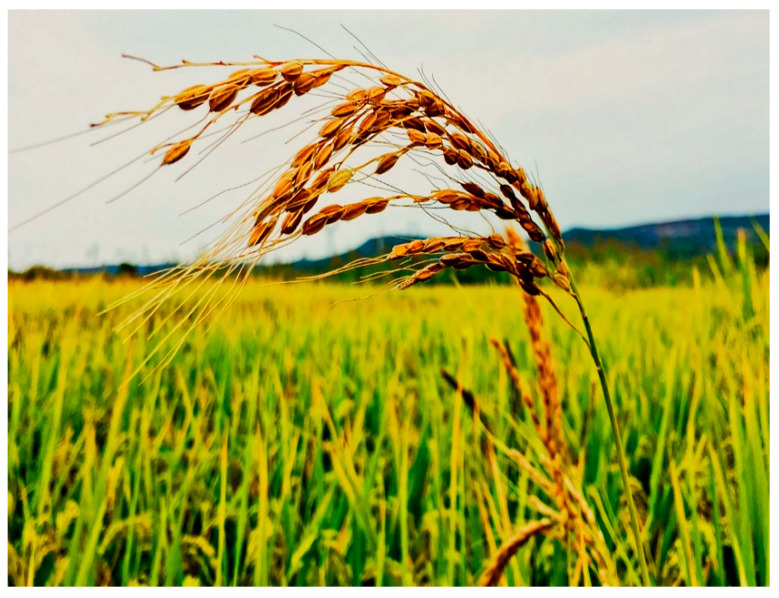
Panicle morphology of weedy rice in a rice field.

Weedy rice (*Oryza sativa* f. *spontanea*, also referred to as red rice, Figure 1) is a noxious weed infesting worldwide rice fields [24,25]. As a conspecific weed, weedy rice belongs to the same biological species of cultivated rice (*O. sativa*), but with strong competition, seed shattering, and prolonged seed dormancy. Consequently, weedy rice is extremely difficult to control, causing great yield and quality losses of cultivated rice [25]. Weedy rice comprises a relatively high level of genetic diversity, probably due to consecutive gene flow and introgression from its co-occurring and diverse rice cultivars, although with extremely low frequencies in each generation (0.008–0.25%) [26]. A previous study including 20 weedy rice populations from northeastern China down to Sri Lanka across a large latitudinal gradient indicates the gradual increases in genetic diversity, estimated by the fingerprints of 20 SSR (simple sequence repeat) loci, with the decreases in latitudes and increases in temperatures [14]. This finding partially supports the hypothesis of increased genetic diversity being associated with higher temperature. However, the conclusion has its limitation to approve the diversity-temperature hypothesis because the increased genetic diversity might also be affected by other factors (e.g., soil types and moisture) across such a large latitudinal span at different sites where the weedy rice materials were collected. Therefore, the most suitable method to test this hypothesis should be generating solid evidence based on the experiments conducted at the same sites with nearly the same environmental conditions but different temperatures.

China has a long history of rice cultivation, and weedy rice infestation of rice fields is a continued problem for rice production [27,28,29]. In the typical tropic rice cultivation regions, such as the Guangdong, Guangxi, and Hainan Provinces, rice is cultivated for two seasons, namely the early- and late rice-cultivation seasons. According to our previous surveys of rice fields in Leizhou of the Guangdong Province, we found abundant weedy rice in rice fields for both seasons [30]. Generally, temperatures between the two seasons are considerably different. Therefore, we believe that such environments with different temperatures and abundant weedy rice populations at the same sites provide an ideal system to study the association of genetic diversity with temperature variation. As an efficient molecular tool to estimate genetic diversity in plant populations, SSR fingerprinting has been extensively used in estimating genetic diversity, differentiation, and structures of weedy rice populations effectively [14,24,31,32,33,34].

The primary objectives of this study are to address the following questions: (1) Is the abundance of genetic diversity in the two-season weedy rice populations affected by temperature differences between the two rice-cultivation seasons? (2) Does genetic differentiation occur between the corresponding early- and late-season weedy rice populations? (3) Is there any correlation between genetic diversity and genetic differentiation in weedy rice populations? The answers to these questions will facilitate our understanding of the relationships between genetic diversity of plant populations and the ambient temperatures, in addition to explaining the possible reasons regarding how genetic diversity can be maintained in plant populations, particularly between the early- and late-season weedy rice populations.

## 2. Materials and Methods 

### 2.1. Plant Materials

A total of 18 weedy rice populations were collected from nine different rice fields in Leizhou, the Guangdong Province of China in 2018 (Supplementary Materials Table S1). The 18 weedy rice populations occurred, respective, in the early- (code as WR-E) and late-season (WR-L) rice cultivation fields in Leizhou. Two populations collected from each field site, corresponding to the early- and late-season rice cultivation fields (with the same lateritic type of soils), were treated as a pair. Consequently, nine population pairs were formed for further comparison (Supplementary Materials Table S1). The spatial distances between the sampled sites of weedy rice population pairs were >5 km. Matured panicles from ~40 individuals were randomly collected to represent each weedy rice population. The distance intervals between weedy rice individuals were >10 m to avoid sampling of similar genotypes. The average duration of the early rice-cultivation seasons was from 1 March to 25 June, with ~141 mm of the monthly precipitation; whereas that of the late rice-cultivation seasons was from 20 July to 5 November, with ~208 mm of the monthly precipitation [35].

### 2.2. DNA Extraction and Polymerase Chain Reaction Amplification

Total genomic DNA was extracted from weedy rice seedlings at about the four-leaf stage, following the CTAB protocol [36]. All rice seedlings were obtained from germinated seeds in a green house in the summer of 2019. Twenty-seven highly polymorphic primer pairs of SSR markers of cultivated rice were selected from the Gramene Database [37] and used as fingerprints (Supplementary Materials Table S2). FAM (blue), ROX (red), or JOE (green) fluorescently labeled the forward primers [32,38]. PCR (polymerase chain reactions) amplification was carried out with a total volume of 10 μL containing 1 μL (20 ng) total genomic DNA, 0.2 μL of 10 mmol/L forward and reverse primer, 0.5 U DNA Taq polymerase, 0.8 μL of 25 mmol/L dNTP, 1 μL of 1 mmol/L reaction buffer with MgCl_2_, and 6.7 μL ddH_2_O in the 2720 Thermal Cycler (Applied Biosystems, Foster, CA, USA). 

The PCR products were separated and analyzed on a capillary electrophoresis analyzer (ABI 3130, Applied Biosystems). Amplified DNA fragments were scored as genotype data (size of the SSR fragments) for each weedy rice sample, owning to the co-dominant feature of the SSR markers [14,32,38,39]. The amplified fragments were scored based on fragment length (bp), using the software GeneMapper version 4.1 (Applied Biosystems).

### 2.3. Estimation of Temperature in the Early and Late Seasons

The data of air temperatures, including the minimum and maximum daily values in Leizhou, were collected from the Tianqi Weather Database [40] from the period of 2011–2020. The 10-year average values of minimum and maximum air temperature were calculated for estimating differences in temperature between the early and late rice-cultivation seasons. The significant differences in air temperature between the early and late rice-cultivation seasons were estimated based on student *t*-test [41].

### 2.4. Estimation of Genetic Diversity

The genotypic data matrix based on the 27 SSR loci of 720 weedy rice individuals representing nine population pairs were analyzed to examine the genetic diversity. The following parameters were calculated: (i) the number of effective alleles per locus (*N_e_*); (ii) the percentage of polymorphic loci (*P*); (iii) Shannon’s information index (*I*, Shannon 1948); and (iv) Nei’s expected heterozygosity (*H_e_*). The analysis of molecular variance (AMOVA) was carried out to estimate the partition of genetic diversity within and among populations, at a level of *p* < 0.001 and 9999 permutations. The statistical analyses were conducted in the software GenAlEx version 6.5 [42].

### 2.5. Analyses of Genetic Divergence and Correlation

The F-statistics (*Fst*, Wright 1978) and number of private alleles (PAS) were computed to estimate genetic differentiation between the early- and late-season weedy rice populations. The statistical analyses were carried out using the software GenAlEx version 6.5 [42]. To estimate the relationship between the abundance of genetic diversity and genetic differentiation among weedy rice populations, correlations of the increased proportion of genetic diversity (Δ*H_e_*) with the *F_st_* values and difference in number of private alleles (ΔPAS) between the early- and late-season weedy rice populations were analyzed, using the software Prism 8 [43] by selecting the method ‘Pearson’. The Δ*H_e_* was estimated based on the calculation of absolute values obtained from *H_e_* of the late-season weedy rice populations subtracting *H_e_* of the corresponding early-season weedy rice populations; whereas the ΔPAS was estimated based on the calculation of absolute values obtained from PAS of the late-season weedy rice populations subtracting PAS of the corresponding early-season weedy rice populations.

### 2.6. Analysis of Genetic Structure

The genetic structure of weedy rice populations was analyzed in the Bayesian clustering algorithm-based program STRUCTURE version 2.3.4 [44] to visualize the genetic component differences between the early- and late-season weedy rice populations. The analysis was based on the SSR genotypic data matrix and the running parameters were set as 100,000 burn-in period, and 200,000 replicates. The admixture model was selected to analyze the genetic components with the correlated allele frequencies [45]. Number of clusters (K) from 2 to 8 were tested with 10 iterations, respectively. The Evanno method was used to detect the number of K groups that best fit the dataset by the Structure Harvester online program [46]. The software CLUMPP version 1.1.2 was used to determine the optimal alignment of the 10 replicates with the ‘Greedy’ algorithm (GREEDY_OPTION = 2, REPEATS = 10,000) [47]. The alignment results were visualized using the software Distruct version 1.1 [48].

## 3. Results

### 3.1. Differences in Air Temperature between the Early and Late Rice-Cultivation Seasons

Analytical results indicated that the 10-year average air temperatures (2011–2020) was about 1.4–1.8 °C higher in the late rice-cultivation seasons than in the early rice-cultivation seasons in the collected region (Table 1). The average differences in air temperature were estimated based on the calculation of the daily climate data (10 years) of Leizhou in the Guangdong Province, China.

The 10-year average values of the maximum and minimum air temperatures in the late rice-cultivation seasons varied between ~30.9 and ~24.0 °C, respectively. However, the 10-year average values of the maximum and minimum air temperatures in the early rice-cultivation seasons varied between ~29.1 and ~22.6 °C, respectively. Noticeably, the 10-year average air temperature at the initial stage of rice growth (seedling) was substantially higher (7.7–8.7 °C) in the late rice-cultivation seasons than in the early seasons (Supplementary Materials Table S3). This observation probably indicated the potential influences of changes in temperatures, which might play a considerable role in weedy rice growth and development at the initial stage.

### 3.2. Genetic Diversity of Weedy Rice Populations and Differentiation between the Early- and Late-Season Populations

The average level of genetic diversity, as indicated by the number of effective alleles (*N_e_*), the percentage of polymorphic loci (*P*), Shannon’s information index (*I*), and expected heterozygosity (*H_e_*), was obviously higher in the late-season weedy rice populations than that in the early-season weedy rice populations (Table 2). This estimation was made based on a total of 720 weedy rice individuals in nine population pairs, using 27 SSR loci as fingerprinting. 

In general, the average levels of these parameters, *N_e_*, *P*, *I*, and *H_e_*, were higher in the late-season weedy rice populations (WR-L, Table 2), although considerable variation in these parameters was observed among the populations. Of the nine population pairs, seven showed higher levels of genetic diversity in the late-season weedy rice populations than those in their corresponding early-season weedy rice populations. Four of these late-season weedy rice populations (Banjiu-L, Dadong-L, Leigao1-L, and Shanwei-L) showed significantly higher levels of genetic diversity than their corresponding early-season populations (Table 2). However, there were two early-season weedy rice populations (Dongcun-E and Leigao2-E) showed a slightly higher level of genetic diversity than their corresponding late-season populations. In addition, the AMOVA result indicated an unexpectedly high proportion of genetic diversity (~80%) within weedy rice populations (Table 3), probably owning to frequent gene flow from their diverse accompanied rice cultivars. Altogether, these results indicated that weedy rice populations infesting the two-season rice fields in Leizhou possessed a relatively high level of within-population genetic diversity, in addition to enhanced genetic diversity in the late-season weedy rice populations.

The results also indicated that the early- and late-season weedy rice populations had considerable genetic differentiation based on the fingerprints generated from 27 SSR loci. The fixation index (*F_st_*) between the corresponding early- and late-season weedy rice populations varied from 0.032–0.103 (Table 4), suggesting the minor to moderate level of genetic differentiation in the population pairs. In addition, the number of private alleles (PAS) was detected in the corresponding early- and late-season weedy rice populations (Table 4). The detected total number of population-specific PAS was 14 in the early-season weedy rice populations, whereas that of population-specific PAS was 25 in the late-season weedy rice populations. The late-season weedy rice populations showed an obviously greater number of population-specific private alleles. Noticeably, one season-specific private allele (RM246-126) was found to be shared by all the late-season weedy rice populations, but no such season-specific private allele was found in the early-season populations. This result may suggest adaptive evolution between the early- and late-season weedy rice populations in different rice-cultivation environments.

### 3.3. Correlation between Genetic Diversity and Differentiation in Weedy Rice Populations

**Figure 2:**
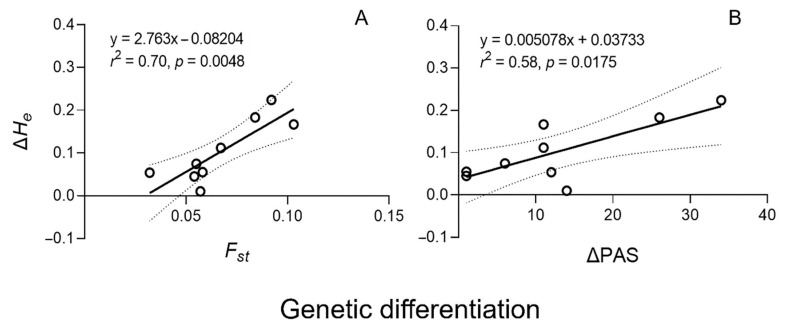
Correlations between Δ*H_e_* (increased proportion of genetic diversity) and *F_st_* (genetic differentiation) (**A**), and between Δ*H_e_* and ΔPAS (differences in number of private alleles) (**B**) of nine pairs of weedy rice populations based on Pearson analysis [41]. Empty circles represent nine pairs of weedy rice populations, solid lines represent linear regression, and dotted lines indicate 95% confidence intervals.

To estimate relationships between genetic diversity and differentiation, we analyzed the correlation between genetic diversity and differentiation of the weedy rice populations from Leizhou. Positive correlations were detected between the increased proportion of genetic diversity (coded as Δ*H_e_*) and genetic differentiation (*F_st_*), as well as between Δ*H_e_* and differences in PAS (ΔPAS) in the early- and late-season weedy rice populations (Figure 2A,B).

A significant positive correlation (*r*^2^ = 0.70, *p* = 0.008) was found between the increased proportion of genetic diversity (Δ*H_e_*) and genetic differentiation (*F_st_*) in the early- and late-season weedy rice populations (population pairs, Figure 2A). In addition, a significant positive correlation (*r*^2^ = 0.58, *p* = 0.0175) was also detected between Δ*H_e_* and the differences in the number of private alleles (ΔPAS) in the corresponding early- and late-season population pairs (Figure 2B). These results probably suggested that a higher level of genetic differentiation, as estimated by *F_st_* and ΔPAS, between the early- and late-season weedy rice populations might give rise to a greater proportion of increased genetic diversity. In other words, the level of increased proportion of genetic diversity between the corresponding early- and late-season weedy rice populations might be closely associated with the level of their genetic differentiation.

### 3.4. Genetic Structure of the Early- and Late-Season Weedy Rice Populations

**Figure 3:**
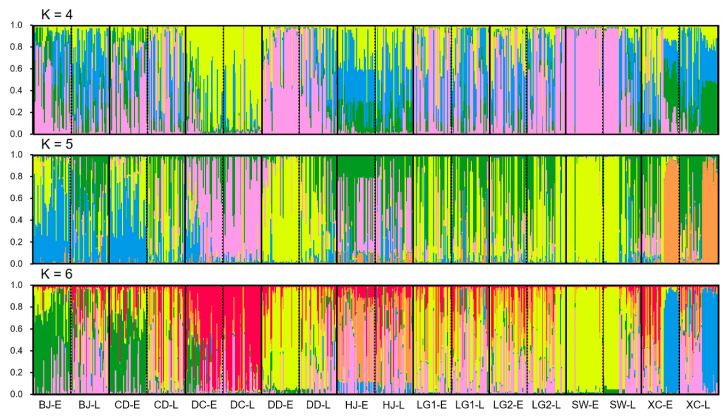
Genetic structure of nine early/late season pairs of weedy rice populations from Leizhou, based on the STRUCTURE analysis of 27 simple sequence repeat (SSR) loci, with the best fit K value (k = 5). The numbers along the vertical axis represent the probability of assignment of the components. Population pairs are isolated by black bold solid lines, and black dotted lines in each population pair separate early- and late-season weedy rice populations. BJ-E and BJ-L, early- and late-season populations at Banjiu; CD-E and CD-L, early- and late-season populations at Chidou; DC-E and DC-L, early- and late-season populations at Dongcun; DD-E and DD-L, early- and late-season populations at Dadong; HJ-E and HJ-L, early- and late-season populations at Hejia; LG1-E and LG1-L, early- and late-season populations at Leigao1; LG2-E and LG2-L, early- and late-season populations at Leigao2; SW-E and SW-L, early- and late-season populations at Shanwei; XC-E and XC-L, early- and late-season populations at Xiachu.

Results from the STRUCTURE analysis exhibited an obvious association between the corresponding early- and late-season weedy rice populations, although these populations showed great variation and considerable degrees of genetic differentiation, as indicated by their complex genetic components (Figure 3). The genetic structure of the weedy rice populations was obtained on the basis of the estimated best fit K-value as 5 according to the Structure-Harvester analysis, in addition to its neighboring K-values of 4 and 6 (Figure 2, Supplementary Materials Figure S1). 

When the K-value was determined as 5 (Figure 3, middle panel), weedy rice demonstrated considerable variation among populations collected from different sites in Leizhou, as illustrated by different genetic components. The corresponding early- and late-season weedy rice populations in the same pairs showed a relatively closer genetic association than those in the other pairs with distinct genetic components. However, the distinct genetic components were also found between some of the early- and late-season population pairs. For example, the BJ-E population possessed a main genetic component (indicated by the blue color), but its corresponding counterpart BJ-L population possessed another main genetic component (indicated by the green color). The similar situation was also found in other pairs of weedy rice populations, such as the CD-E vs. CD-L, DD-E vs. DD-L, and SW-E vs. SW-L populations (Figure 3, middle panel). When the K-value was determined as 4 and 6, the early- and late-season weedy rice populations demonstrated comparable patterns in terms of the genetic structures illustrated by their genetic components (Figure 3, upper and lower panels).

In addition, genetic structures of the weedy rice populations showed an obvious admixture in terms of the genetic components in nearly all weedy rice populations, no matter the K-value was determined as 4 or 5 or 6 (Figure 3, upper, middle, and lower panels). These results suggested strong genetic introgression of the weedy rice populations, most likely from the rice cultivars. Noticeably, a generally more complicated pattern of the genetic structures was detected in many of the late-season weedy rice populations, which was apparently associated with their higher level of within-population genetic diversity. 

## 4. Discussion

### 4.1. Increased Genetic Diversity of Weedy Rice Associated with Higher Ambient Temperature

Our results based on the molecular fingerprints of 27 SSR loci clearly indicated a greater level of genetic diversity in weedy rice populations occurring in the late-season rice fields with nearly 2 °C higher temperature than that in the early-season rice fields, although two early-season weedy rice populations showed a slightly higher level of genetic diversity than their two corresponding counterparts occurring in the late seasons. The unexpected results could be explained by the histories of rice cultivation and emergence of weedy rice in those particular fields, where the accumulation of genetic variation might not be sufficient to exhibit an increased level of genetic diversity in the late-season weedy rice populations. Nevertheless, the average data from all populations used in this study demonstrated a generally higher level of genetic diversity in the late-season weedy rice populations. 

In addition, our results from the genetic structure analysis in this study also demonstrated a more complicated pattern of genetic components in many weedy rice populations occurring in the warmer seasons for late rice cultivation. These results also support our conclusion that higher temperature promotes the creation of greater genetic diversity from another angle. Likewise, similar patterns regarding the association between more complicated genetic structure and genetic diversity were detected in other plant species, such as in cultivated rice [49], wild rice [50], and maize [51]. Altogether, these results support our findings regarding the higher level of genetic diversity in the late-season weedy rice populations. 

The increased level of genetic diversity is more likely associated with the higher average air temperature in the late-season rice fields. In other words, the ambient temperature can considerably affect the abundance of genetic diversity in weedy rice, probably also in other plant species [18]. This conclusion is based on the fact that the major difference between the two-season rice fields at the same sites was the higher temperature (~2 °C) in the late rice-cultivation seasons. Particularly, the difference in the average air temperature (~9 °C) in the rice seedling period was more pronounced between the two seasons, which may have a role in the creation of greater genetic diversity through accelerating metabolic and mutation rates at the initial stage for the growth and development of rice. Previous studies already indicated that the higher ambient temperature can affect the metabolic and mutation rates of organisms, resulting in more abundant genetic diversity [4,52]. In addition, higher ambient temperature can accelerate molecular-evolutionary speed of organisms [53], also affecting the abundance of genetic diversity. Given that the major, if not only, difference in environmental conditions for weedy rice populations occurring at the same sites was the temperature between the two seasons, it is reasonable to suggest based on results from this study that higher ambient temperature is likely one of the important ecological factors promoting genetic diversity, although this conclusion needs further investigations to approve. 

The above finding may address the first question raised in the Introduction section regarding increased genetic diversity in weedy rice that is associated with the higher temperature. The hypotheses about the formation of biodiversity (including genetic diversity) in association with temperature, such as the latitudinal biodiversity gradient model were proposed [4,22,52,53]. However, these hypotheses were proposed based on the global scale, where many ecological factors, except for temperature, may also affect the formation of genetic diversity. Also, Wang et al. (2019) [14] found a large latitudinal gradient for genetic diversity in weedy rice populations from northeastern China down to Sri Lanka, and supported the hypothesis of increased genetic diversity to be associated with higher temperature. Similarly, we believe that other environmental factors such as soil types, amount of precipitation and rice farming styles at different sites across such a large scale also have influences on genetic diversity. 

Results from the above studies may have not provided sufficient evidence to approve the diversity-temperature hypothesis at a small scale. In contrast, findings provided by this study are based on the experimental assessment of genetic diversity of early- and late-season weedy rice populations occurring in exactly the same rice fields. At the same sites, many ecological factors are the same, but temperatures are different between the early and late rice-cultivation seasons. Therefore, the results obtained from this study provided direct and relatively solid evidence for the hypothesis that the higher ambient temperature can promote the formation of genetic diversity, although other factors also play important roles.

Weedy rice is a strictly self-pollinating taxon [31,54]. Usually, self-pollinating species are expected to have relatively low level of genetic diversity, particularly within-population genetic diversity [55,56,57]. However, we detected unexpectedly high level of genetic diversity of weedy rice in this study, particularly within populations (80%, based on AMOVA). The relatively high level of genetic diversity found within weedy rice populations is probably due to historically accumulated allele exchange through gene flow and introgression from different rice cultivars adopted at different period of time, although the same varieties or landraces were commonly used in the early- and late-season rice fields at the same period of time [58]. The admixture found in genetic structure of the weedy rice populations in this study and other studies [14,31,32,34] supported introgression of weedy rice populations from rice cultivars. Similarly, weedy rice populations from other studies [14,32,34] and other self-pollinating plant species such as cultivated barley [59], and the forage grass *Elymus* species [60] were also reported to have an unexpectedly higher level of genetic diversity owning to pollen- or seed-mediated gene flow. 

### 4.2. Limited Gene Flow Promotes the Maintenance of increased Genetic Diversity in the Late-Season Weedy Rice Populations

Our results further indicated considerable genetic differentiation between most of the corresponding early- and late-season weedy rice populations, based on both the fixation index (*F_st_*) and differences in the number of private alleles (ΔPAS). This finding clearly demonstrates limited gene flow between the early- and late-season weedy rice populations. Limited gene flow between weedy rice populations is commonly reported, as indicated by *F_st_* values or the mixed mating models generated form molecular fingerprints [14,31,61,62]. Also, limited crop-to-weed gene flow (0.008–0.25% per generation) was observed between rice cultivars and weedy rice populations, as indicated by the pollen-mediated gene flow experiments [26,54,63,64,65]. All these findings confirm the strictly self-pollination mating system of weedy rice, causing limited gene flow [31,54]. Therefore, our finding regarding genetic differentiation between the corresponding early- and late-season weedy rice populations is reasonable. In other words, weedy rice populations occurring in the early- and late-seasons at the same field sites are not identical in terms of their genetic components. Namely, there are two cryptic early- and late-season populations in the same rice field. This conclusion is first reported from this study.

Generally, limited gene flow can effectively reduce the exchange of genetic materials, causing genetic heterogeneity of plant populations. In contrast, excessed gene flow can increase the exchange of genetic materials, causing genetic homogeneity of plant populations. Consequently, the observed phenomenon of genetic heterogeneity between the early- and late-season weedy rice populations may cause the reduced exchange of genetic materials, which may play an important roles in maintaining the increased proportion of genetic diversity formed in the late-season weedy rice populations. It is therefore interesting to understand the relationships between genetic differentiation and the increased proportion of genetic diversity (Δ*H_e_*) in the studied weedy rice populations. As expected, significant positive correlations were detected between genetic differentiation, as represented by the fixation index (*F_st_*) and differences in the number of private alleles (ΔPAS), and the increased proportion of genetic diversity (Δ*H_e_*). In other words, the observed high proportion of increased genetic diversity might be maintained in the late-season weedy rice populations by limited gene flow between the weedy rice populations occurring in different seasons at the same field sites. 

All results from this study approved our expectation that the relatively higher level of genetic diversity maintained in the late-season weedy rice populations is probably caused by the limited gene flow between the early- and late-season weedy rice populations. Previous studies generally indicated that limited gene flow causes genetic divergence or differentiation of plant populations [66,67,68], although sufficient gene flow was observed to cause genetic homogeneity [69]. In addition, those studies also indicated that the maintenance of genetic diversity requires the presence of stronger genetic barriers to restrict gene exchanges between populations to resist the homogenization effect caused by gene flow [70,71,72,73,74]. Results from all the previous studies support our expectation that limited gene flow would help to maintain newly formed genetic diversity in plant populations. 

Based on our study, we propose a hypothesis—the maintenance of genetic diversity, no matter high or low, formed in the early- and late-season weedy rice populations depends on limited gene flow between these populations. This is probably true to other plant populations/species with limited gene flow. For example, Sagnard et al. (2011) [75] provided an evidence of low level of genetic differentiation (*F_st_* = 0.037) of Sorghum (*Sorghum bicolor*) in the cultivated gene pool and wild gene pool among different climatic zones. As a result, no obvious differences in genetic diversity (*H_e_*, 0.530–0.606) were detected within cultivated gene pool and wild gene pool. However, the authors detected a high level of genetic differentiation (*F_st_*=0.40) among different racial sorghum types, and consequently, considerable differences in genetic diversity (*H_e_*, 0.325–0.614). These findings clearly suggested that the limited gene flow, as indicated by genetic differentiation, played a certain role in maintaining genetic diversity within Sorghum populations. In addition, Jacquemyn et al. (2010) [76] also reported the limited gene flow between populations of a rare thistle (*Cirsium acaule*) in the calcareous grassland area, which has played an active role in maintaining a low level of genetic diversity within populations. Likewise, evidence was also found in many other plant species, such as the tropical tree *Copaifera langsdorffii* Desf [77] and walnut (*Juglans regia*) [78]. Therefore, we propose a hypothesis that limited gene flow may play a considerable role in maintaining genetic diversity created during the evolutionary process of plant populations. Whether this hypothesis is a general pattern to explain the formation and maintenance of genetic diversity needs further approval based on more population genetic and evolution studies.

## 5. Conclusions

In this study, we found a relatively high level of genetic diversity in the late-season weedy rice populations exposed to slightly higher ambient temperatures compared with the early-season populations from the same field sites with lower temperatures. This finding provided direct experimental evidence to support the diversity-temperature hypothesis in which the higher ambient temperature can promote greater genetic diversity. We also found considerable genetic differentiation between the corresponding early- and late-season weedy rice populations collected from the same fields, probably caused by limited gene flow. The positive correlation between the increased proportion of genetic diversity and genetic differentiation suggests a low level of the exchange of genetic materials, which probably acts as a factor to maintain genetic diversity within the early- or late-season weedy rice populations during the evolutionary processes. Whether or not the richness and maintenance of genetic diversity in plant populations/species follow such underlying mechanisms still needs further investigations.

## Figures and Tables

**Table 1:** The 10-year (2011–2020) average values of the minimum and maximum air temperatures in early and late rice-cultivation seasons in Leizhou in the Guangdong Province, China (Numbers in parentheses following averages indicate standard deviation, SD).

Year	Minimum Temperature (°C)		Maximum Temperature (°C)	
	Early ^1^	Late	Early	Late
2011	20.6 (4.51)	23.5 (2.12)	27.3 (5.82)	30.5 (2.59)
2012	22.5 (4.15)	23.5 (2.12)	28.8 (5.19)	30.4 (2.32)
2013	22.2 (3.26)	23.3 (2.32)	28.8 (3.95)	30.0 (2.03)
2014	22.6 (3.56)	23.8 (2.19)	28.7 (4.88)	31.0 (2.19)
2015	23.0 (3.49)	24.0 (2.27)	30.1 (4.59)	30.8 (2.72)
2016	22.7 (4.14)	25.1 (1.60)	29.1 (5.06)	31.8 (2.24)
2017	22.9 (3.41)	24.9 (2.67)	29.0 (4.03)	31.8 (2.60)
2018	22.8 (3.65)	23.4 (2.35)	29.4 (3.89)	30.3 (1.93)
2019	23.3 (3.04)	24.3 (2.20)	29.9 (3.74)	31.3 (2.25)
2020	22.9 (3.64)	24.0 (2.43)	29.8 (4.79)	30.6 (2.94)
**10-year average**	**22.6 (0.75)**	**24.0 (0.62)**	**29.1 (0.80)**	**30.9 (0.62)**
*p*-value ^2^	<0.001		<0.001	
Difference ^3^	1.4		1.8	

^1^ Early: the early rice cultivation season from 1 March to 25 June; Late: the late rice cultivation season from 20 July to 5 November. ^2^ Comparison was made between the 10-year average values of the minimum and maximum air temperatures of early and late rice-cultivation seasons using the Student *t*-test [41]. ^3^ Difference in the 10-year average values of temperature between the early and late rice-cultivation seasons.

**Table 2:** Genetic diversity parameters in nine weedy rice population pairs for comparison from the early and late rice-cultivation seasons in Leizhou in the Guangdong Province, China, based on 27 simple sequence repeat (SSR) loci (Numbers in parentheses following averages indicate the standard error, SE).

Location	Population	*N_e_* ^a^	*P*/%	*I*	*H_e_*
Banjiu	Banjiu-E ^b^	1.78 (0.14)	88.90	0.66 (0.09)	0.35 (0.05)
	Banjiu-L	**2.36 (0.15)**	**96.30**	**0.93 (0.08)**	**0.51 (0.04)**
Chidou	Chidou-E	1.79 (0.15)	96.30	0.69 (0.08)	0.37 (0.04)
	Chidou-L	1.92 (0.12)	96.30	0.77 (0.07)	0.42 (0.04)
Dongcun	Dongcun-E	2.14 (0.18)	96.30	0.84 (0.07)	0.47 (0.04)
	Dongcun-L	1.84 (0.12)	96.30	0.72 (0.07)	0.40 (0.04)
Dadong	Dadong-E	1.26 (0.07)	85.19	0.31 (0.06)	0.16 (0.03)
	Dadong-L	**1.64 (0.12)**	**92.59**	**0.65 (0.07)**	**0.35 (0.04)**
Hejia	Hejia-E	1.88 (0.14)	92.59	0.71 (0.08)	0.40(0.04)
	Hejia-L	2.07 (0.15)	96.30	0.82 (0.07)	0.45 (0.04)
Leigao1	Leigao1-E	1.54 (0.11)	88.89	0.57 (0.07)	0.31 (0.04)
	Leigao1-L	**1.95 (0.14)**	**96.30**	**0.77 (0.07)**	**0.42 (0.04)**
Leigao2	Leigao2-E	1.73 (0.13)	92.59	0.69 (0.07)	0.37 (0.04)
	Leigao2-L	1.60 (0.09)	92.59	0.61 (0.06)	0.32 (0.04)
Shanwei	Shanwei-E	1.09 (0.03)	70.37	0.15 (0.03)	0.07 (0.02)
	Shanwei-L	**1.52 (0.09)**	**96.30**	**0.56 (0.06)**	**0.29 (0.03)**
Xiachu	Xiachu-E	2.32 (0.16)	96.30	0.93 (0.07)	0.51 (0.04)
	Xiachu-L	2.51 (0.22)	96.30	1.00 (0.08)	0.52 (0.04)
Average	WR-E ^c^	1.73 (0.13)	89.71 (0.03)	0.62 (0.08)	0.33 (0.05)
	WR-L	1.93 (0.11)	95.47 (0.01)	0.76 (0.05)	0.41 (0.03)

^a^*N_e_* = No. of effective alleles per locus; *p* = Percentage of polymorphic loci; *I* = Shannon’s information index (Shannon, 1948); *H_e_* = expected heterozygosity. ^b^ E indicates the early season, L indicates the late season. ^c^ WR-E, weedy rice populations in the early rice-cultivation seasons; WR-L, weedy rice populations in the late rice-cultivation seasons. Bolded numbers indicate significant differences between the early- and late-season weedy rice populations at *p* < 0.05 level based on student *t*-test [41].

**Table 3:** Results of AMOVA (analysis of molecular variance) from 18 weedy rice populations based on 27 simple sequence repeat (SSR) loci (*p* < 0.001, 9999 permutations).

Source	d.f.	SS	MS	Est. Var.	%
Among populations	17	2030	119.428	1.42	20%
Within populations	1422	8123	5.712	5.71	80%
Total	1439	10153		7.13	100%

d.f., degrees of freedom; SS, sum of squared deviations; MS, mean of squared deviations; Est. var., variance component estimates; %, percentage of total variation.

**Table 4:** Number of private alleles (PAS) and genetic differentiation (*F_st_*) of nine pairs of weedy rice populations from early and late rice-cultivation seasons in Leizhou in the Guangdong Province, China, based on 27 simple sequence repeat (SSR) loci (Numbers in parentheses indicate the standard error, SE).

Locations	Population Pairs	No. of PAS	Average Freq. of PAS	Season-Specific PAS	*F_st_*
Banjiu	Banjiu-E ^a^	17	0.19 (0.05)	/	0.103
	Banjiu-L	28	0.14 (0.06)	/	
Chidou	Chidou-E	23	0.13 (0.06)	/	0.058
	Chidou-L	22	0.11 (0.04)	/	
Dongcun	Dongcun-E	19	0.10 (0.05)	/	0.055
	Dongcun-L	13	0.16 (0.09)	/	
Dadong	Dadong-E	9	0.16 (0.07)	/	0.084
	Dadong-L	35	0.09 (0.03)	/	
Hejia	Hejia-E	12	0.05 (0.02)	/	0.032
	Hejia-L	24	0.06 (0.02)	/	
Leigao1	Leigao1-E	17	0.09 (0.03)	/	0.067
	Leigao1-L	28	0.10 (0.05)	/	
Leigao2	Leigao2-E	17	0.07 (0.02)	/	0.054
	Leigao2-L	18	0.11 (0.07)	/	
Shanwei	Shanwei-E	3	0.33 (0.17)	/	0.092
	Shanwei-L	37	0.08 (0.03)	/	
Xiachu	Xiachu-E	9	0.11 (0.04)	/	0.057
	Xiachu-L	23	0.06 (0.01)	/	
Average	WR-E ^b^	14 (2.1)	0.14 (0.03)	/	0.067
	WR-L	25 (2.5)	0.10 (0.01)	RM246-126 ^c^	

^a^ E indicates the early season, L indicates the late season. ^b^ WR-E, weedy rice populations in early rice-cultivation seasons; WR-L, weedy rice populations in late rice-cultivation seasons. ^c^ RM246-126, seasonal-specific private allele among all WR-L, locate in SSR locus RM246.

## Data Availability

Not applicable.

## Supplementary Materials

The following are available online at https://www.mdpi.com/2079-7737/10/2/71/s1, Figure S1: Delta-K value of each K (2–8) was used to determine the appropriate estimated numbers of the genetic components (K values) by the STRUCTURE HARVESTER online program. True number of populations (K) is often identified using the maximal value of Delta-K [46]. Table S1: Locations of nine pairs of weedy rice populations each containing 40 individuals collected from Leizhou in the Guangdong Province, China. Table S2: The 27 simple sequence repeat (SSR) primer pairs used in this study with detail information on their DNA sequences and motifs. Table S3: The daily average minimum and maximum temperature at different growth stages of cultivated rice in the early and late rice-cultivation seasons in Leizhou from 2011 to 2020.
