# Supplementary material for: Increases in Genetic Diversity of Weedy Rice Associated with Ambient Temperatures and Limited Gene Flow
Source: Biology (Basel). 2021 Jan 20;10(2):71. doi: 10.3390/biology10020071 (PMC7909424; doi:10.3390/biology10020071)
Supplement: Supplementary file 1 [file biology-10-00071-s001.zip › Supplementary Materials/Supplementary Materials Table S1.docx]

| Collecting site | Code of  population pairs | Population ID | GPS location |
| --- | --- | --- | --- |
| Banjiu | BJ | BJ-E ^1^ | 20°50.3056′ N, 109°55.1151′ E |
|  |  | BJ-L |  |
| Chidou | CD | CD-E | 20°52.4986′ N, 109°46.5007′ E |
|  |  | CD-L |  |
| Dongcun | DC | DC-E | 20°46.0530′ N, 110°03.5190′ E |
|  |  | DC-L |  |
| Dadong | DD | DD-E | 20°46.4718′ N, 110°07.4522′ E |
|  |  | DD-L |  |
| Hejia | HJ | HJ-E | 20°56.2696′ N, 109°58.8599′ E |
|  |  | HJ-L |  |
| Leigao1 | LG1 | LG1-E | 20°48.3371′ N, 110°11.2522′ E |
|  |  | LG1-L |  |
| Leigao2 | LG2 | LG2-E | 20°49.5160′ N, 110°10.3570′ E |
|  |  | LG2-L |  |
| Shanwei | SW | SW-E | 20°52.0350′ N, 110°01.0780′ E |
|  |  | SW-L |  |
| Xiachu | XC | XC-E | 20°49.5480′ N, 110°05.1190′ E |
|  |  | XC-L |  |

**Table S1.** Locations of nine pairs of weedy rice populations each containing 40 individuals collected from Leizhou in Guangdong Province, China

^1^ E indicates the early season, L indicates the late season.
