# Supplementary material for: Increases in Genetic Diversity of Weedy Rice Associated with Ambient Temperatures and Limited Gene Flow
Source: Biology (Basel). 2021 Jan 20;10(2):71. doi: 10.3390/biology10020071 (PMC7909424; doi:10.3390/biology10020071)
Supplement: Supplementary file 1 [file biology-10-00071-s001.zip › Supplementary Materials/Supplementary Materials Table S2.docx]

**Table S2.** The 27 simple sequence repeat primer pairs used in this study with detail information on their DNA sequences and motifs

| Primer ID | Chromosome location | Forward primer | Reverse primer | Repeat motif |
| --- | --- | --- | --- | --- |
| RM1 | 1 | GCGAAAACACAATGCAAAAA | GCGTTGGTTGGACCTGAC | (GA)_26_ |
| RM246 | 1 | GAGCTCCATCAGCCATTCAG | CTGAGTGCTGCTGCGACT | (CT)_20_ |
| RM9 | 1 | GGTGCCATTGTCGTCCTC | ACGGCCCTCATCACCTTC | (GA)_15_GT(GA)_2_ |
| RM154 | 2 | ACCCTCTCCGCCTCGCCTCCTC | CTCCTCCTCCTGCGACCGCTCC | (GA)_21_ |
| RM208 | 2 | TCTGCAAGCCTTGTCTGATG | TAAGTCGATCATTGTGTGGACC | (CT)_17_ |
| RM262 | 2 | CATTCCGTCTCGGCTCAACT | CAGAGCAAGGTGGCTTGC | (CT)_16_ |
| RM282 | 3 | CTGTGTCGAAAGGCTGCAC | CAGTCCTGTGTTGCAGCAAG | (GA)_15_ |
| RM3525 | 3 | ACACTCTCAGCTCATCAAGACC | GGGCAAGTGGTCAAATCTTG | (CT)_33_ |
| RM514 | 3 | AGATTGATCTCCCATTCCCC | CACGAGCATATTACTAGTGG | (AC)_12_ |
| RM3524 | 4 | CTGTCTCCGTCTTCCTCACTCG | TGGAGAAATCTCCCTTCCTGAGC | (CT)_31_ |
| RM470 | 4 | TCCTCATCGGCTTCTTCTTC | AGAACCCGTTCTACGTCACG | (CTT)_14_ |
| RM169 | 5 | CACCTCCTCCAAGATCCTTATGC | CTCTCTGTCTCGCTGTCTGTTGC | (GA)_12_ |
| RM3575 | 5 | ACAGCCTCAAATTGTGAGCAAGG | GCTGTATGATCTGTATCCATCCATCC | (GA)_12_ |
| RM480 | 5 | GCTCAAGCATTCTGCAGTTG | GCGCTTCTGCTTATTGGAAG | (AC)_30_ |
| RM19424 | 6 | TTCAACGCTAGCTCTTGATGACG | GTGATCATTTGTGCTCATCTGTGG | (AAAG)_5_ |
| RM345 | 6 | ATTGGTAGCTCAATGCAAGC | GTGCAACAACCCCACATG | (CTT)_9_ |
| RM469 | 6 | AGCTGAACAAGCCCTGAAAG | GACTTGGGCAGTGTGACATG | (AG)_15_ |
| RM11 | 7 | TCTCCTCTTCCCCCGATC | ATAGCGGGCGAGGCTTAG | (GA)_17_ |
| RM560 | 7 | GCAGGAGGAACAGAATCAGC | AGCCCGTGATACGGTGATAG | (CT)_12_ |
| RM149 | 8 | GCTGACCAACGAACCTAGGCCG | GTTGGAAGCCTTTCCTCGTAACACG | (AT)_10_ |
| RM22254 | 8 | GTATAAGCCACATGGCGGTTTAGG | GAAGTTGACGCCCTGAAGAAGC | (ATAG)_5_ |
| RM223 | 8 | GAGTGAGCTTGGGCTGAAAC | GAAGGCAAGTCTTGGCACTG | (CT)_25_ |
| RM215 | 9 | CAAAATGGAGCAGCAAGAGC | TGAGCACCTCCTTCTCTGTAG | (CT)_16_ |
| RM23662 | 9 | GAGAGGACGATGGCACTATTGG | CGAGGAACTTGATTCGCATGG | (GGC)_10_ |
| RM258 | 10 | TGCTGTATGTAGCTCGCACC | TGGCCTTTAAAGCTGTCGC | (GA)_21_(GGA)_3_ |
| RM286 | 11 | GGCTTCATCTTTGGCGAC | CCGGATTCACGAGATAAACTC | (GA)_16_ |
| RM12 | 12 | TGCCCTGTTATTTTCTTCTCTC | GGTGATCCTTTCCCATTTCA | (GA)_21_ |
