# Supplementary figures and images for: Increases in Genetic Diversity of Weedy Rice Associated with Ambient Temperatures and Limited Gene Flow
Source: Biology (Basel). 2021 Jan 20;10(2):71. doi: 10.3390/biology10020071 (PMC7909424; doi:10.3390/biology10020071)

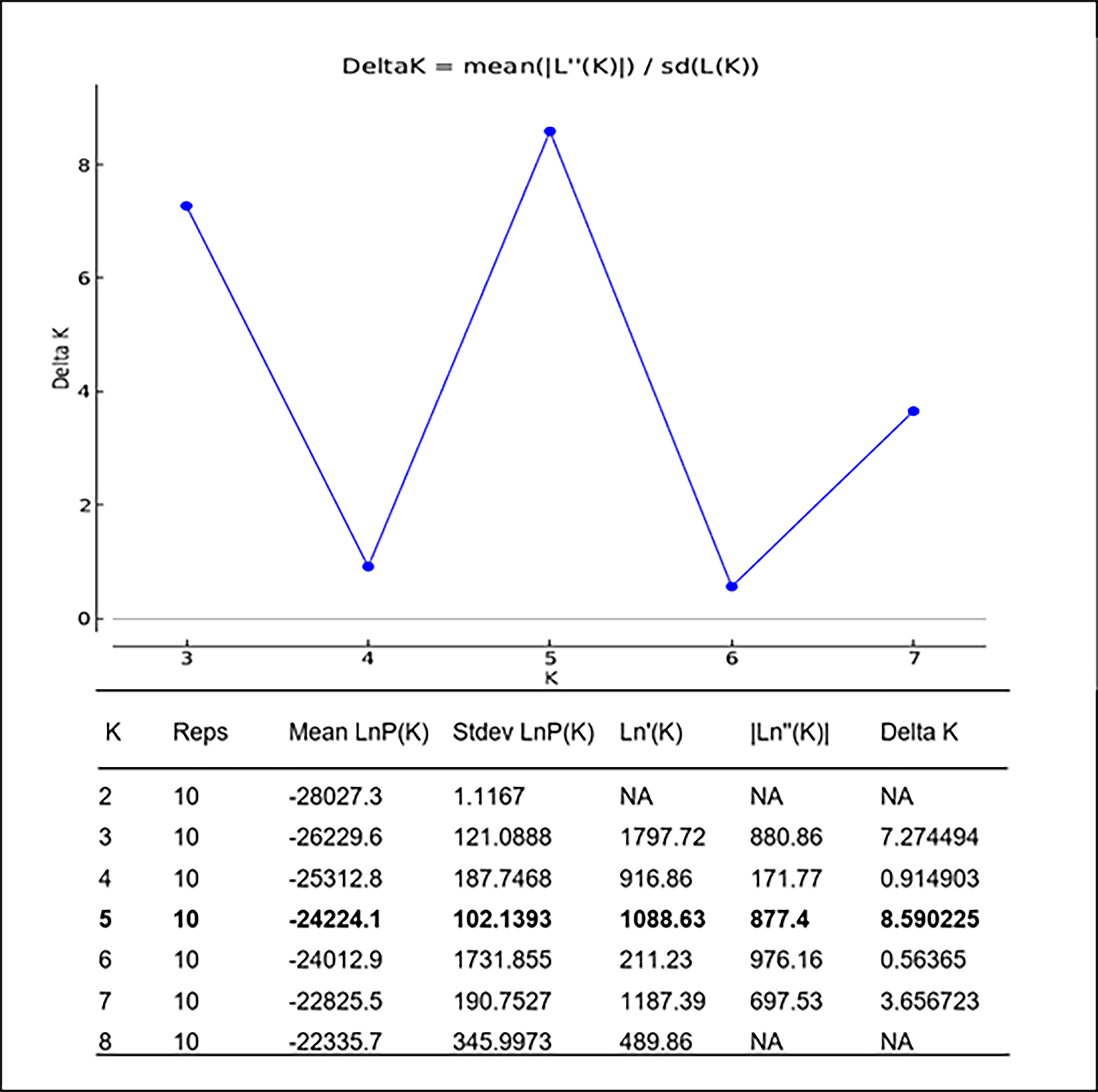

Supplement: Supplementary file 1 [file biology-10-00071-s001.zip › Supplementary Materials/Supplementary Materials Figure S1.tif]
